# The longitudinality of care from the perspective of Family Health users

**DOI:** 10.1590/1980-220X-REEUSP-2024-0051en

**Published:** 2024-09-13

**Authors:** Anna Maria Meyer Maciel, Angelina Lettiere-Viana, Silvana Martins Mishima, Tauani Zampieri Fermino, Silvia Matumoto

**Affiliations:** 1 Universidade do Estado de Minas Gerais, Unidade Acadêmica de Passos, Passos, MG, Brazil.; 2 Universidade de São Paulo, Escola de Enfermagem de Ribeirão Preto, Departamento de Enfermagem Materno-infantil e Saúde Pública, Ribeirão Preto, SP, Brazil.

**Keywords:** Primary Health Care, Continuity of Patient Care, Patients, Atención Primaria de Salud, Continuidad de la Atención al Paciente, Pacientes

## Abstract

**Objective:**

To analyze longitudinality in the production of care in Family Health from the perspective of users.

**Method:**

Qualitative research carried out with 18 users of a family health unit in a municipality in the state of São Paulo. The data was produced through semi-structured interviews and the empirical material was analyzed by interpreting the meanings in the light of the theoretical framework of continuity of care and longitudinality.

**Results:**

22 ideas were identified and grouped into three meanings: organization and operationalization of work in the family health unit, self-care and the health system. The first highlighted elements of organizational constraints, workforce, hard and soft technologies. The second direction pointed to the user’s co-responsibility for their health condition and lifestyle, making it possible to recognize longitudinality as: discontinuous or focused and continuous or extended. And in the third meaning, the understanding of the functioning of the three levels of care was presented with structural and technological demarcations.

**Conclusion:**

The users recognized potential and weaknesses in the three meanings referring to the constituent elements of the theoretical framework. Family Health is capable of offering continuous or extended longitudinality, even in a municipality with low coverage of the strategy. However, this scenario can weaken the process of developing the attribute from this perspective, as it limits access to other levels of care and compromises its structuring elements and dimensions and, consequently, the continuity of care.

## RESUMEN

**Objetivo:**

Analizar la longitudinalidad en la producción de cuidados en Salud de la Familia desde la perspectiva de los usuarios.

**Método:**

Investigación cualitativa realizada con 18 usuarios de una unidad de Salud de la Familia de un municipio del interior de São Paulo. Los datos se produjeron por medio de entrevistas semiestructuradas y el material empírico se analizó interpretando los significados a la luz del marco teórico de la continuidad del cuidado y de la longitudinalidad.

**Resultados:**

Se identificaron 22 ideas agrupadas en tres significados: organización y operacionalización del trabajo en la unidad familiar de salud, autocuidado y sistema de salud. La primera destacó elementos de limitaciones organizativas, fuerza de trabajo, tecnologías duras y blandas. El segundo sentido apuntó a la corresponsabilidad del usuario por su condición de salud y estilo de vida, permitiendo reconocer la longitudinalidad como: discontinua o focalizada y continua o extendida. Y en el tercer sentido, la comprensión del funcionamiento de los tres niveles de atención se presentó con demarcaciones estructurales y tecnológicas.

**Conclusiones:**

Los usuarios reconocieron potencialidades y debilidades en los tres sentidos referidos a los elementos constitutivos del marco teórico. Salud de la Familia es capaz de ofrecer longitudinalidad continua o extendida, incluso en un municipio con baja cobertura de la estrategia. Sin embargo, este escenario puede debilitar el proceso de desarrollo del atributo desde esta perspectiva, ya que limita el acceso a otros niveles de atención y compromete sus elementos estructurantes y dimensiones y, en consecuencia, la continuidad de la atención.

## INTRODUCTION

Primary Health Care (PHC) is a care model corresponding to the first level of care as well as the main gateway to health systems, seeking to respond to the most common needs of a population. Its operationalization takes into account economic, political and social arrangements in different development scenarios. The first discussions to define the functions of PHC began in the 1900s after the establishment of the English national health insurance scheme, which proposed restructuring the care model according to levels of technological density and treatment costs, based on the attributes of first contact, comprehensiveness, coordination of care and longitudinality^(1)^.

The main expression of PHC in Brazil is Family Health (FH) which, in 2020, covered 133,710,730 people in all regions, a 62.62% population coverage, with 43,286 teams, in addition to the basic health units that provide health care for the rest of the population. The FH contributes to universal access to health and seeks to implement the attributes of PHC through the work of a multi-professional team in health promotion, prevention, rehabilitation and surveillance actions in the territories with assigned populations^(2)^. The organization, expansion and consolidation of the FH as the main representation of a solid PHC can occur in different ways and of different magnitudes in the municipalities, depending on population size, human development index^(3)^ and political interest. PHC is part of a health system made up of secondary and tertiary levels with actions and services of medium and high technological density. In this system, referral to other levels of care is directly related to continuity of care (COC)^(4)^.

Longitudinality derives from longitudinal, which means dealing with the growth and changes of individuals or groups over a period of years. It presupposes a long-term personal relationship between the same health team and service users that can be interrupted for any reason, without discontinuing the relationship. It allows the team to develop a certain familiarity with users and their families which facilitates: recognition of problems of various kinds, early diagnosis of illnesses, preventive care, assertiveness in clinical diagnosis, drug prescription and routine follow-up. At the same time, it can contribute to: a reduction in hospital admissions and overall treatment costs; and an increase in user satisfaction and confidence in the COC^(1)^.

Contemporarily in Brazil, the terms COC and longitudinal bond have been used synonymously with longitudinality. They have similar meanings, although they are different expressions^(5)^. In the early 2000s, a hierarchical conceptual definition of COC was proposed, taking into account user records, the types and location of establishments providing actions and services, interpersonal relationships between professionals and users, family members and all those involved in the production of care^(6)^. In this way, it was established that the COC admits informational continuity (organized, systemic and available collection of users’ medical and social information), longitudinal/chronological continuity (place of reference where care is traditionally produced and received) and interpersonal continuity (relationship of trust and responsibility between users and professionals). In other words, a minimum of informational continuity is necessary for chronological continuity to be present through interpersonal continuity^(6)^. In this context, the term longitudinal bond is defined as a therapeutic relationship established between the user and the professionals of a team in a health unit, which is a regular source of care over time^(7)^.

There are three dimensions to the attribute of longitudinality^(7)^: the existence and recognition of a regular source of primary care, the establishment of a lasting therapeutic bond between users and the professionals in a team, and the continuity of recording information about the service user’s health problem. It can be seen that these dimensions are aligned with the constituent elements of COC in an USA study^(6)^ which proposed: longitudinal/chronological, interpersonal and informational continuity, respectively.

Various instruments are used to assess COC at the international level: continuity of care index, number of providers used, sequential continuity index, continuity probability index, sequential continuity probability index, among others^(6)^. In Brazil, longitudinality and the other attributes of PHC have been assessed using specific instruments, such as the Primary Care Assessment Tool for children and adult patients, with the aim of evaluating various aspects from the perspective of those receiving the care. Cross-sectional and population-based studies have already applied this quantitative tool in various regions, both in family health units (FHU) and basic health units^(3,8,9)^.

In municipalities in Rio Grande do Sul^(3)^ and Minas Gerais^(8)^, users evaluated longitudinality as fragile in terms of interpersonal continuity, related respectively to: the lack of recognition of the user’s integrality, which led to a limited production of care, and the lack of follow-up with the same professionals in a team, which interfered with the development of trust between them. In a municipality in the state of Mato Grosso^(9)^, the attribute was rated satisfactorily, demonstrating COC through the building of lasting therapeutic bonds between users and professionals.

Understanding that longitudinality permeates COC and is an essential attribute for the development and consolidation of a strong and stable PHC, this study aims to find out how users perceive longitudinality and what COC represents in maintaining their health. In this way, the qualitative dimension, exploring the experiences of users in this study, is what sets it apart from those that have studied the subject using evaluation instruments. Thus, the aim of this study is to analyze longitudinality in the production of care in the FH from the users’ point of view.

## METHOD

### Type and Site of the Study

This is a descriptive and analytical study with a qualitative approach, inspired by the criteria of the Consolidated Criteria for Reporting Qualitative Research instrument, carried out with users of two FH teams (FHt) in the municipality of Ribeirão Preto-SP, with 703,293 inhabitants, of which only 165,600 or 25% of the population are followed up by 51 FHt, distributed in 22 FHU, the rest being followed up by Primary Care teams^(10)^.

### Population and Selection Criteria

Initially, the municipal PHC coordinators were contacted, requesting the indication of two FHts organized in their minimum configuration, with their members (doctor, nurse, nursing technicians/auxiliaries and community health workers) working together for at least two years, since this is the average length of time that professionals remain in the same team^(11)^. Once three FHts had been nominated, one refused to take part in the study on the grounds that its members were being restructured, while the other two agreed to take part and were accepted as the setting for the investigation. These FHts were located in the same FHU and were briefed on the research process. At the time of the study, each FHt had four Community Health Workers (CHWs) and one CHW from each FHt was away from work with their respective micro-areas uncovered.

In order to cover the entire area of the FHU, each of the eight CHWs at the FHU was asked to nominate two users from their area and one from each uncovered micro-area, observing the following as inclusion criteria: adults over the age of 18 who were cognitively able to answer the questions and who had been following up with the team for at least five years^(1)^, enough time for users to establish longitudinal links with the PHC team. The participants were selected by convenience sampling, and there were no refusals to take part in the interviews.

### Data Collection

Two approaches were made to the users indicated by the CHWs: a telephone contact to invite and schedule the interview and the second to conduct the interview in their homes or at the FHU, according to their preference. The semi-structured interviews were carried out by the first author during the month of May 2023, in the morning and afternoon, on weekdays, and lasted a maximum of 28 minutes. The interviews were recorded with authorization and guided by a guide that investigates the dimensions of longitudinality in Brazil^(7)^, shown in Chart 1. The questions in the guide underwent minor language adaptations to make them easier for users to understand, for example: “1. Do you think the unit has an assigned population or a population for care within a region?” The content of the interviews was sufficient to meet the objectives of the study and there was no need to include more participants.

**Chart 1:** Questions for investigating the dimensions of the longitudinality attribute in Brazil^(7)^ – Rio de Janeiro, 2011.

Identification or recognition of the regular source of care
1. Does the unit have client enrollment? 2. Is the unit recognized by the assigned population as a place of care for old and new health problems?
**Interpersonal relations**
3. Is the patient seen regularly by the same doctor and/or nurse for routine appointments? 4. Do the professionals who see the patient know the patient’s family and social history? 5. If in doubt about treatment, can the patient speak to the professional who sees them regularly? 6. Is there enough time during treatment for patients to explain their doubts, complaints and concerns? 7. Are the patient’s doubts, concerns and complaints valued? Are they recorded in the medical records? 8. Does the professional express themselves clearly, in a way that the patient understands? 9. Is there room for the patient to discuss their treatment and make decisions together with the professional?
**Informational continuity**
10. Do health professionals use medical records when providing care? 11. Are health professionals informed about all the medicines used by the patient? 12. Are healthcare professionals informed about the tests carried out on the patient? 13. In the event of a referral to a specialist, does this professional receive recorded information from the unit that sees the patient on a regular basis? 14. In the case of referrals for specialist consultations or external examinations, are the results returned to the clinician who sees the patient on a regular basis? 15. Are health professionals informed when a patient is unable to obtain the prescribed medication?

**Source:** National study^(7)^.

### Data Analysis and Processing

The interviews were transcribed by a specialized firm, and their content, once checked, was analyzed in terms of the relationships between the parties, interpreting the meanings of the statements to understand the users’ view of the research topic, anchored in the methodological framework of the interpretation of meanings. This framework is part of the comprehensivist currents of the social sciences - which are dedicated to analyzing words, groups, institutions, contexts and their relationships - and advances in interpretation beyond the content, considering the context and logic produced in a given culture and society^(12)^. It was developed in three stages: exploration of the material with the impregnation of the statements; definition of ideas seeking to reveal the implicit beyond the explicit, interpreting and problematizing them^(12)^ and the search for broader meanings that articulated the objective of the study, the theoretical-conceptual framework and the empirical data. In order to present the ideas extracted from the analysis of the empirical material, an interpretative matrix was constructed, taking up the constituent elements of the COC and the dimensions of longitudinality in PHC in order to highlight similarities and differences, especially in the sense that it encompassed a larger and more diverse number of ideas, as shown in Chart 2 in the results section.

**Chart 2:** Interpretative matrix of the research according to the
methodological framework^(12)^, containing the three broadest
meanings and their respective ideas.

Organization and operationalization of work at the FHU
1. Family Health Unit catchment area
2. Family Health Unit’s limited service’s capacity
3. Family Health Unit work process
4. CHW functions
5. Longitudinal care by the team
6. Work tools (Health records)
7. Professional turnover in the team
8. Priority of care at the Family Health Unit
9. Disease-centered approach to production of care
10. Expanded view of care production
11. Lack of closeness between professionals and users
12. Trust and bonds
13. Empathy and dialogue
14. Assertive communication
15. FHU quality of care
**Organization and operationalization of self-care**
16. Users’ co-responsibility for their health
17. Users’ knowledge about their care
**Organization and operation of the health system**
18. Referral and counter-referral
19. Limited service’s capacity at secondary level
20. Health system organization
21. State’s responsibility with regard to high-cost drugs
22. Limitations on the supply of essential medicines

**Source:** Authors.

### Ethical Aspects

The study complied with the ethical standards for research with human beings, in accordance with Resolution 466/2012, and was cleared by the Research Ethics Committee under opinion number 5.933.046/2023. All participants previously signed an informed consent form. In order to preserve anonymity, participants were identified using an alphanumeric code (letter E followed by an ordinal number).

### Data Availability

The entire set of anonymized data supporting the results of this study was made available in February 2024 in the ScIELO Data research data repository, the preliminary version of which is available at: https://data.scielo.org/dataset.xhtml?persistentId=doi:10.48331/scielodata.6JB0YH.

## RESULTS

Among the 18 participants, 14 were women and 4 were men, aged between 30 and 87. In terms of level of education, eight had incomplete primary education, five had completed primary education, four had completed secondary education and one had completed tertiary education. Half of the participants reported being retired or on a pension, seven were home workers, one reported being a doorman and one was a human resources assistant. The majority have lived in the area covered by the FHU for more than ten years, one for less than ten years and one couldn’t say.

After analyzing the empirical material, 22 ideas about longitudinality in the production of care in the FH were identified and grouped into three broader meanings, ordered according to a logic of understanding and conformation of the essential conditions for strengthening and guaranteeing COC and longitudinality in PHC, related respectively to: the organization and operationalization of work in the FHU, self-care and the health system, as shown in Chart 2.

### Organization and Operationalization of Work at the Family Health Unit

In the first meaning, the interviewees shared elements about the organizational restrictions (area of coverage and limited capacity for medical care), the workforce (composition and attribution of FHt members, their competencies and skills to produce care), hard technologies (instruments for producing care) and soft technologies (trust, bond, empathy, dialogue, assertive communication and lack of intimacy with the user):

*I think they have a catchment area, because I’ve seen a lot of people ask that they wanted to be cared for and told that they couldn’t, because they have to be located in the neighborhood, so they have an area that they attend to (E16).*

*This issue of an open agenda is also a bit complicated when it comes to getting an appointment (doctor). Because, most of the time, they say that they don’t have an open schedule, so you have to go every day to see if there is an open schedule (...). I made an appointment with (nurse’s name) and it was very quick. To do the Pap smear was very quick. But with (name of doctor), it’s a bit more difficult. Because she sees a lot of people. She’s very patient. It’s like this, you get there, it’s always full, crowded (E10).*

*We have to go in to get weighed, measured and our blood pressure checked (E5).*

*The pre-consultation girls, you talk to them. If I need to talk to him (the doctor), I go through them. Maybe they can talk to him, give me some feedback, you know? (E15).*

*The nurse, when you get there, not only does she put you at ease so you can say everything you have the right to say... I mean, you have a way of saying everything you want to her and telling her everything, even private things. And she welcomes you very well and doesn’t say: Come on, there’s another patient waiting.... So she gives you all the support you need. That’s why she sets a very different time for each patient (E8).*

*Every time we go for a consultation, he (the doctor) takes all the medical records and reads them to us (E9).*

*We usually talk not only about illness problems, if you have a child who is ill or unemployed, I have a son who found out three years ago that he had cancer, so if you ask the doctor, she can tell you our story... you start to have a real family service, she knows things. If she comes to the house, she already asks about everyone, she knows my children’s names, so I think it’s not just the physical, she has a family life (E16).*

*Oh, I discuss, I ask. Like psychotherapy. I say: Why do you think I need to do it? It’s because you have that little nest problem, what’s it like? Nest abandonment... Empty nest syndrome. I have it because the children have left, there’s no one here anymore, just me and this sister. She (the doctor) felt it when I went to talk about the children, she feels that I’m a bit hurt, because I’m here alone, but it’s not their fault... Then the doctor knows everything and she said: I’m going to send you there, do you accept? She doesn’t force me to do anything. She gave me a piece of paper, I took it to the counter and started. I mean. She understands me (E2).*

*They’ve already learned our neighborhood language here, they speak our language, so you can understand them and me (E2).*

*Doctor Z was a wonderful doctor, he’s a guy (nurse’s name). He absorbed your particular problems and told you his too, which is the hardest thing in the world. Doctor Y, if you don’t look here at reception, you don’t even know his name properly; and that one didn’t (...). He knew every patient by name. That’s not the case today, you know? (E8).*

In this sense, there was a closer relationship with interpersonal continuity or the establishment of a lasting therapeutic bond between FHt professionals and users, and with longitudinal/chronological continuity or the existence and recognition of a regular source of primary care. These elements and dimensions were experienced respectively by users to the extent that they identified FHt members as reference subjects for the implementation of necessary and timely health practices, with the Family Health Unit being the place determined for this purpose.

### Organization and Operationalization of Self-Care

In the second meaning, the interviewees highlighted their co-responsibility for their health condition and lifestyle, emphasizing self-care (practicing and maintaining healthy habits), self-knowledge (about their illnesses and possible complications) and socialization, as the fragments indicate:

*I belong to a group of people, up to 97 years old, who practice sport. I run and throw weights. I train two or three times a week. Maybe on the 20th we’ll go to (name of city) to compete. There are various things, but in my case, it’s running and throwing. So that’s what we do: we train there a lot. I put almost the palm of my hand on the floor, with my legs stretched out. Before we do anything, we do a lot of gymnastics, and then we go training, running, a whole series of things (E4).*

*I only take thyroid medication, I don’t have a blood pressure problem; my blood pressure is 11 over 8, 12 over 8. I don’t let it go up. Pap smears, mammograms, I already go for them, he (doctor Z) tells me to do them, I go and do them (E9).*

*Before the pandemic, we had parties, Mother’s Day, Father’s Day, Festa Junina (...). It’s end-of-year parties, get-togethers, then we set up a club, spend the day at the club, make Secret Santa... Whoever goes gets a present. It’s not expensive, it’s a little souvenir. Then we draw lots and do it, spend the day. Everyone brings a dish, pie, cake, whatever they want. With the staff at the post, we have all this (...). We have a barbecue; everyone brings something and spends the day at the club (E5).*

In this sense, the active stance of users in managing their own lives in search of well-being and quality of life was highlighted, taking responsibility for self-care associated with the relevance of social interaction facilitated by the extramural actions organized by the Family Health Unit, an idea that converges with interpersonal continuity or the establishment of a lasting therapeutic bond between professionals and Family Health Unit users. At the same time, it can be inferred that in this meaning, the above element and dimension implicitly bring a close relationship to the existence and recognition of a regular source of primary care, given that various group meetings are organized to share festive dates throughout the year to help promote health.

### Organization and Operation of the Health System

In this third meaning, the interviewees shared their understanding of the functioning of the three levels of care, recognizing their structural and technological demarcations (referral and counter-referral, limitations in secondary care capacity associated with restrictions in the computerized system and in the supply of basic and specialized medicines) respectively as evidenced in the fragments:

*You can be treated (at the Family Health Unit) but, if it’s an emergency, we’re always advised to go to the UPA (Emergency Care Unit), but here we have consultations (...) a diabetes that will treat, the team doctor will give medication, a future treatment (...) (E11).*

*If a new case appears (they treat it). Then, if they can’t treat it, it’s referred to the specific doctor, for example, if it needs to go to orthopedics, neuro, geriatrician. So, when new cases come up, they try to solve them, if they can’t treat them, they refer them to the place that... to the specific area (E16).*

*I’ve never heard anything about whether they (the doctors who attend at secondary level) send feedback to the doctor (the FHt doctor). I don’t think so. If I’m treated there, then it’s still there, I don’t know if it’ll come back here afterwards, it’ll just be a piece of paper, I don’t know (E6).*

*I need an ophthalmologist and I’ve been wanting to make an appointment for a while. I can’t get an appointment. They gave me the phone (...). Sometimes it’s switched off, sometimes it’s busy, sometimes it rings and there’s no answer (...). I haven’t been to the ophthalmologist for about two and a half years. I’m already feeling it, because I drive and at night, I can’t drive any more, because my glasses aren’t working much anymore (E2).*

*The professionals here are working at the top of their game, because sometimes they can’t afford to... they get paid to pass on to us. They do everything they can (...). Sometimes, for example, there’s no medicine at the clinic, why? Because the Health Department hasn’t sent the medicine, you know? (...) This post here does everything it can for me. If they don’t have medicine here, they call the other health center so I can go and get it (E12).*

*It happens that I can’t find the medicine, there are some types of medicine that they draw up a document for me to pick up at the Hospital das Clínicas. It’s... I don’t know what, it’s high-priced (...). I went there and they didn’t have the medicine that was on the document. Almost 20 days later, I had the medicine that we call to find out if it’s available (E4).*

In this sense, the empirical data indicates that Family Health Unit users are aware of the organization of the health system into levels of care, associating this experience with longitudinal/chronological continuity or the existence and recognition of the Family Health Unit as a regular source of primary care, describing difficulties in accessing other services to which they are referred in due course. In addition, they attest to the responsibility of the municipal level in the supply and availability of certain essential health products. Information continuity or the continuity of recording information about the user’s health problem seems to be limited to each of the services used, and there is no articulation of clinical data between them.

## DISCUSSION

The sociodemographic characterization of those interviewed, the majority of whom were women and elderly people with a low level of education, working in informal occupations, reflects the demographic transition index for each social segment from the 1980s to 2010, determined by a gender gap in life expectancy at birth attributed to the increase in deaths from violent causes, which mostly affected men. In addition, an older age profile with a reduction in fertility levels, due to Brazil’s socio-economic transformations, has directly influenced female reproductive behavior. In addition, a new profile of people has been incorporated into the current consumer market: the lower middle class with an income of up to two minimum wages – a massive consumer of actions, goods and services – thanks to inclusive public social policies aimed at transferring income^(13)^.

In addition to this scenario of demographic transition over the last three decades, there is also the trajectory of population ageing, which by 2030 will reach a ratio of 76 elderly people for every 100 young people. This projection has sparked a discussion about health care, which will involve resizing the supply of actions and services in this sector - most of which are still geared towards treating acute conditions and exacerbations of chronic conditions - taking into account the chronic non-communicable diseases of the elderly population, as well as the costs associated with their respective treatments^(13)^. In this logic of producing care that polarizes health events, the power of longitudinality, developed by the FHt, stands out, as it can support more effective, efficient and efficient monitoring of the set of morbidities inherent to this population segment, which is growing in longevity every year.

With regard to the organization and operation of work at the Family Health Unit, the restrictions on the supply of medical care pointed out by users were also evidenced in other studies^(8,14-16)^ which highlighted the delay between scheduling a medical appointment and the appointment itself. This can lead to complications in users’ chronic health conditions, since regular and frequent consultations in PHC represent access and continuity of care provided by the team, and are directly related to: users’ satisfaction with the service, their quality of life, adherence to proposed treatments, health indicators, reduced hospitalization costs^(17)^ and fewer referrals to other levels of care^(4)^.

Users recognize that nurses play a leading role in organizing the production of care at the Family Health Unit, which involves planning and defining users’ access to the actions and services needed for each case, promoting COC. This active stance strengthens the element of interpersonal continuity^(6)^ or the establishment of a lasting therapeutic bond between professionals and users^(7)^, resolves conflicts and manages situations in everyday care, directly influencing the team’s work process^(14,18,19)^.

Users’ degree of affiliation is also centred on the doctor, who is recognized as their reference professional^(9,19)^. However, users who do not identify the name of the doctor who accompanies them in PHC can develop fragile interpersonal relationships with the team which can interfere with access and continuity of care^(20)^.

Although some users say that valuing biopsychosocial aspects (capillarized by soft technologies) contributes to more comprehensive care, the complaint-diagnosis-conduct triad is also criticized and portrayed as a lack of dialogue and a plastering of curative actions^(16,19,21)^. In the world of soft technologies, the clarity of the technical guidelines given by health professionals also constitutes assertive communication, one of the elements that make up effective longitudinality^(16)^.

Users seem to know that there is a hard technology that organizes and systematizes their clinical data so that the FHt and other health professionals at other levels of care can prescribe care; however, they were not aware of the importance of the flow of this information between services. The use of physical medical records has been replaced by computerized systems that allow access to users’ administrative, social and clinical information, even if it is fragmented or has been produced by different professionals and service providers. For this hard technology to express the element of informational continuity^(6)^ or the dimension of continuity in the recording of information about the user’s problem^(7)^, it is necessary to organize the data so that it is available in a timely manner for the teams at the different levels of care to produce more comprehensive, articulated and integrated care. On the other hand, different operational systems used by different health services that are not connected make it difficult to develop longitudinal care^(17,18,22,23)^, especially for patients with chronic health conditions who require more regular care^(24,25)^.

According to users, the organization of the flow of care within the service is limited, leading to the segmentation of care^(16)^, when each professional uses their technical and scientific expertise, records the information in the medical record and refers the user to the next professional who will add their knowledge in a similar way, until the doctor arrives - a common practice in the Family Health Unit in the municipality where the research was carried out, as a result of the technical and social division of health work.

Regarding the element of interpersonal continuity or the dimension of establishing a lasting therapeutic bond between professionals and users, reproduced by soft technologies, users showed that the welcoming attitude of the team expressed through cordiality, active listening, dialog, attention, empathy, affection and accountability contribute to building bonds between users and workers, promoting user satisfaction with the work offered at the Family Health Unit and adherence to therapeutic plans to control chronic health conditions^(15,26)^. Users’ trust in the team is an essential concept that predisposes them to greater involvement and personal commitment to their own health^(27)^. These skills can favor team members’ familiarity with users and their families, to the extent that they are willing to get to know their problems and anxieties, answer their questions and needs^(27)^. Therefore, based on the premise of creating a bond through strong interpersonal ties and cooperation, the realization of longitudinal care, at different stages of life, makes it possible to value the broad conception of the health-disease process and generate satisfaction with the care received in PHC^(21)^.

Regarding the organization and operationalization of self-care, the user’s co-responsibility for their health condition and lifestyle can be associated with the production of qualified longitudinality in two ways: the first is characterized by a response to a specific or one-off demand translated into discontinuous or focused longitudinality, which is constituted in the element of longitudinal/chronological continuity^(6)^ or in the dimension of the existence and recognition of the Family Health Unit as a regular source of primary care^(7)^ sought after each new health event or problem. The second is characterized by continuous or extended longitudinality expressed by a frequent, lasting and safe response to control, for example, a chronic health condition that depends on building the element of interpersonal continuity^(6)^ or the dimension of establishing a lasting therapeutic bond with the FHt^(7)^.

The concept of producing extended and focused care related to PHC attributes was initially discussed^(28)^ in the first decade of the 2000s, at the time of the more rapid and comprehensive implementation of the FH in Brazil. This debate generated a classification of the attribute of comprehensive care, taking into account its different meanings^(28)^, which have been widely studied in the practice of FHt care. In this second decade of sluggish development of the FH, as a result of various movements to dismantle PHC and the Unified Health System as a whole, presenting a qualification for another attribute, longitudinality, based on international^(6)^ and national^(7)^ theoretical-conceptual references, can be considered an act of resistance to the production of primary care and the credibility of the FHT work process.

Still on the subject of self-knowledge and self-care, in general Family Health Units develop some kind of collective activity associated with health education organized by the team, such as lectures, physical activity, dance, games, handicrafts, choir or others that enable the elderly to socialize and occupy themselves^(15)^, since many of them have no support network or family presence. On the international scene, similar collective and individual initiatives that stimulate, lead and uniquely guide changes in the habits of PHC users are also being developed by multidisciplinary teams^(27)^. This movement can be compared to the work of the Family Health Support Nuclei which, integrated into the FHt, focus on health promotion and disease prevention actions - although they have lost ground with the reorganization of PHC funding^(29)^.

Health promotion actions with a focus on active and healthy ageing and the organization of extramural collective activities, mentioned by the interviewees, make it possible to share experiences, reduce social isolation and strengthen interpersonal ties. In this way, an important component of PHC is put into practice: the production of care through an expanded view of the health-disease process that occupies space in the hegemonic model centered on the disease^(25)^. Furthermore, valuing users’ autonomy and active participation in making decisions related to their health also helps to strengthen the element of interpersonal continuity or the dimension of establishing a lasting therapeutic bond between users and professionals, contributing to COC and longitudinality^(27)^.

As for the third meaning of the organization and operation of the health system, the structural and technological demarcations recognized by users can be compared to other studies in that the counter-referral also proved to be fragile, with no return to the primary level of the referred user’s care process^(14,18,21,22,30)^. In this reality, we can infer that the element of informational continuity^(6)^ or the dimension of continuity in recording information about the user’s health problem^(7)^ has been broken.

This way of operating referrals and counter-referrals intensifies the fragmentation of care and suggests that the professionals at the different levels of care are less accountable, compromising their effectiveness. In addition, the lack of vacancies at the secondary and tertiary levels leads to isolated and non-interactive care, keeping the focus of attention solely on the demand that led to the referral^(8,14,18)^. In addition, referrals for specialized consultations, more complex exams and surgeries are the result of the slowness of the healthcare network^(8,15,22,30)^.

The lack of medicines was pointed out by users as one of the problems with the organization and operation of the health system. Thus, the lack of essential medicines is an aspect that can weaken the production of care and result in users being discredited and dissatisfied with the Family Health Unit and the way the health network works, causing them to seek out other health establishments to have their demand met or to wait for local recovery^(14-16)^. This situation can contribute to a lack of adherence to the treatment proposed by the team, as users don’t always have the financial means to buy the prescribed medication, even if it is basic and strategic^(31)^. Similarly, user access to the Specialized Component of Pharmaceutical Assistance in municipalities, which is the responsibility of the state, is hampered by the lack of medicines and information for users, bureaucracy in dispensing and delays in acquiring certain drugs^(32)^.

The study’s limitations lie in the fact that the users interviewed were the ones who most often went to the Family Health Unit and took care of their health, given that the population was mostly made up of women, which may have influenced some of the results. Furthermore, the qualitative data portrays the reality of two FHts in a municipality with low FH coverage and high professional turnover, which makes it difficult to generalize the findings. It is therefore recommended that further studies be carried out to qualitatively analyze COC and longitudinality in other PHC settings and contexts.

The benefits of this research include the identification and discussion of the constituent elements of COC and the dimensions of longitudinality from the perspective of users, their relevance and their operationalization.

## CONCLUSION

FH users recognized longitudinality through a structural, technological and organizational demarcation, identifying the workforce in the production of care implemented by soft and hard technologies, sharing weaknesses and potentialities. They admit what they need, want or understand as necessary to maintain and treat their health, both in relation to the organization of the system as a whole, and in relation to what goes on in the Family Health Unit and, more particularly, in relations with the FHt or a specific worker and their responsibility in this process.

Longitudinality is an attribute of PHC that is under permanent construction in order to guarantee COC. It depends on the macro-political organization of a health network with timely actions and services. At the same time, it depends on encounters in living work in act, in other words, in the micro-politics of its production, since the people who operate the machine called the health system are people - workers and users.

The FH can offer continuous or extended longitudinality. However, low coverage of the strategy can compromise the development of this attribute from this perspective, insofar as it limits access to actions, services and health professionals at other levels of care and, consequently, to COC. As COC and longitudinality involve informational continuity (or the recording of information about the user’s problem), longitudinal/chronological continuity (or the existence and recognition of a regular source of primary care) and interpersonal continuity (or the establishment of a lasting therapeutic bond between professionals and users), it is imperative to develop continuity in the management of these processes, which are intertwined in the coordination of the healthcare network, considering the nuances of population ageing.

## References

[1] Starfield B. (2002). Atenção primária: equilíbrio entre necessidades de saúde,
serviços e tecnologia.. Brasília (DF): UNESCO, Ministério da Saúde;.

[2] Brasil. (2020). Ministério da Saúde. Secretaria de Atenção Primária à Saúde.
Histórico de Cobertura da Atenção Básica [Internet].. Brasília (DF): Ministério da Saúde;.

[3] Kessler M, Lima SB, Weiller TH, Lopes LF, Ferraz L, Eberhardt TD (2019). Longitudinalidade do cuidado na atenção primária: avaliação na
perspectiva dos usuários. Acta Paul Enferm.

[4] Olthof M, Groenhof F, Berger MY (2019). Continuity of care and referral rate: challenges for the future
of health care. Fam Pract.

[5] Giovanella L, Mendonça MHM. (2012). Políticas e sistemas de saúde no Brasil..

[6] Saultz JW. (2003). Defining and measuring interpersonal continuity of
care. Ann Fam Med.

[7] Cunha EM, Giovanella L (2011). Longitudinalidade/continuidade do cuidado: identificando
dimensões e variáveis para a avaliação da Atenção Primária no contexto do
sistema público de saúde brasileiro. Cien Saude Colet.

[8] Fagundes LC, Lima CFQ, Dias LC, Fortes MAM, Silveira AAD, Fagundes DC (2021). Avaliação dos atributos essenciais da atenção primária em Montes
Claros – MG. Bionorte.

[9] Masochini RG, Farias SNP, Sousa AI (2022). Avaliação dos atributos da Atenção Primária à Saúde na
perspectiva dos idosos. Esc Anna Nery.

[10] Ribeirão Preto. (2021). Prefeitura da Cidade;.

[11] Tonelli BQ, Leal APR, Tonelli WFQ, Veloso DCMD, Gonçalves DP, Tonelli SQ (2018). Rev Fac Odontol.

[12] Gomes R., Minayo MCS, Deslandes SF, Gomes R (2016). Análise e interpretação de dados de pesquisa qualitativa.
In:. organizadores. Pesquisa social: teoria, método e criatividade.
Petrópolis: Vozes;.

[13] Oliveira ATR, ONeill MMVC (2013). Fundação Oswaldo Cruz, organizador. A saúde no Brasil em 2030:
prospecção estratégica do sistema de saúde brasileiro: população e perfil
sanitário [Internet].

[14] Silva CTS, Assis MMA, Espíndola MMM, Nascimento MAA, Santos AM (2021). Desafios para a produção do cuidado na Atenção Primária à
Saúde. Rev Enferm UFSM.

[15] Trintinaglia V, Bonamigo AW, Azambuja MS (2022). Equipes de Saúde da Família e Equipes de Atenção Primária:
avaliação do cuidado segundo a ótica da pessoa idosa. Saúde Redes.

[16] Carvalho BLR, Boeck GA, Back IR, Santos AL (2023). Análise da assistência prestada na atenção primária e fatores
associados na perspectiva de idosos diabéticos. Rev Baiana Saúde Pública.

[17] Ha NT, Harris M, Preen D, Robinson S, Moorin R (2019). A time-duration measure of continuity of care to optimise
utilisation of primary health care: a threshold effects approach among
people with diabetes. BMC Health Serv Res.

[18] Miotello M, Koerich C, Lanzoni GMM, Erdmann AL, Higashi GDC (2020). Atuação do enfermeiro na consolidação do cuidado longitudinal às
pessoas com doença arterial coronariana. Rev Enferm UFSM.

[19] Acylino EM, Almeida PF, Hoffmann LMA (2021). Acesso e continuidade assistencial na busca por cuidado em saúde:
tecendo a rede entre encontros e entrelaços. Physis.

[20] Leniz J, Gulliford MC (2019). Continuity of care and delivery of diabetes and hypertensive care
among regular users of primary care services in Chile: a cross-sectional
study. BMJ Open.

[21] Além KFS, Peixoto IVP, Monteiro EC, Rodrigue WCC, Pacífico MOS, Ferreira BWR (2022). Longitudinalidade na Atenção Primária à Saúde: um estudo
bibliométrico. Res Soc Dev.

[22] Rojas Manzano KL, Toro Delgado N, Eraso Riascos DJ, Mondragón-Sánchez EJ. (2022). Rev Cuid.

[23] Villalon GE. (2021). Continuidad del cuidado. Evid Actual Pract Ambul.

[24] Ribeiro SP, Cavalcanti MLT (2020). Atenção Primária e Coordenação do Cuidado: dispositivo para
ampliação do acesso e a melhoria da qualidade. Cien Saude Colet.

[25] Schenker M, Costa DH (2019). Avanços e desafios da atenção à saúde da população idosa com
doenças crônicas na Atenção Primária à Saúde. Cien Saude Colet.

[26] Lachtim SAF, Freitas GL, Lazarini WS, Marinho GL, Horta ALM, Duarte ED (2023). Vínculo e acolhimento na Atenção Primária à Saúde:
potencialidades e desafios para o cuidado. Tempus.

[27] Sagsveen E, Rise MB, Grønning K, Westerlund H, Bratås O (2019). Respect, trust and continuity: a qualitative study exploring
service users’ experience of involvement at a Healthy Life Centre in
Norway. Health Expect.

[28] Pinheiro R, Mattos RA, Cecílio LCO. (2009). organizadores. Os sentidos da integralidade na atenção e no cuidado à
saúde..

[29] Brasil. (2020). Ministério da Saúde. Secretaria de Atenção Primária à Saúde.
Departamento de Saúde da Família. Nota técnica nº 3/2020-DESF/SAPS/MS, de 28
de janeiro de 2020.. Dispõe sobre o Núcleo Ampliado de Saúde da Família e Atenção Básica
(NASF-AB) e Programa Previne Brasil [Internet]..

[30] Ollé-Espluga L, Vargas I, Mogollón-Pérez A, Soares-de-Jesus RPF, Eguiguren P, Cisneros AI (2021). Care continuity across levels of care perceived by patients with
chronic conditions in six Latin-American countries. Gac Sanit.

[31] Barreto RMA, Albuquerque IMAN, Cunha ICKO, Freitas CASL, Braga JCT, Lima RBS (2020). Avaliação da dimensão estrutura para a qualidade da atenção
primária à saúde. Enferm Foco.

[32] Brito AH, Araujo MO (2022). Percepção dos usuários sobre o acesso a medicamentos do
componente especializado da assistência farmacêutica. HU Rev.

