# Supplementary material for: The longitudinality of care from the perspective of Family Health users
Source: Rev Esc Enferm USP. 2024 Sep 13;58:e20240051. doi: 10.1590/1980-220X-REEUSP-2024-0051en (PMC11412057; doi:10.1590/1980-220X-REEUSP-2024-0051en)
Supplement: Translation

# Translation: A longitudinalidade do cuidado sob perspectiva dos usuários da Saúde da Família

**DOI:** 10.1590/1980-220X-REEUSP-2024-0051pt

**Authors:** Anna Maria Meyer Maciel, Angelina Lettiere-Viana, Silvana Martins Mishima, Tauani Zampieri Fermino, Silvia Matumoto

**Affiliations:** 1 Universidade do Estado de Minas Gerais, Unidade Acadêmica de Passos, Passos, MG, Brasil.; 2 Universidade de São Paulo, Escola de Enfermagem de Ribeirão Preto, Departamento de Enfermagem Materno-infantil e Saúde Pública, Ribeirão Preto, SP, Brasil.

**Keywords:** Atenção Primária à Saúde, Continuidade da Assistência ao Paciente, Pacientes

## Abstract

**Objetivo:**

Analisar a longitudinalidade na produção do cuidado na Saúde da Família sob a ótica dos usuários.

**Método:**

Pesquisa qualitativa realizada com 18 usuários de uma unidade de saúde da família de um município do interior paulista. Os dados foram produzidos por meio de entrevista semiestruturada e o material empírico foi analisado por meio da interpretação dos sentidos à luz do referencial teórico da continuidade do cuidado e longitudinalidade.

**Resultados:**

22 ideias foram identificadas e agrupadas em três sentidos: organização e operacionalização do trabalho na unidade de saúde da família, do cuidado de si e do sistema de saúde. No primeiro, foram destacados elementos sobre restrições organizacionais, força de trabalho, tecnologias duras e leves. O segundo sentido apontou a corresponsabilidade do usuário pela sua condição de saúde e estilo de vida, permitindo reconhecer a longitudinalidade qualificada em: descontínua ou focalizada e contínua ou ampliada. E no terceiro, foi apresentado o entendimento sobre o funcionamento dos três níveis de atenção com demarcações estruturais e tecnológicas.

**Conclusão:**

Os usuários reconheceram potencialidades e fragilidades nos três sentidos referentes aos elementos constituintes do referencial teórico. A Saúde da Família é capaz de oferecer uma longitudinalidade contínua ou ampliada, mesmo em um município com baixa cobertura da estratégia. Entretanto, esse cenário pode enfraquecer o processo de desenvolvimento do atributo nessa perspectiva, na medida em que limita o acesso aos demais níveis de atenção e compromete seus elementos e dimensões estruturantes e consequentemente a continuidade do cuidado.

## INTRODUÇÃO

A Atenção Primária à Saúde (APS), enquanto modelo assistencial, corresponde ao primeiro nível de atenção ou à principal porta de entrada dos sistemas de saúde que busca responder às necessidades mais comuns de uma população. Sua operacionalização considera arranjos econômicos, políticos e sociais nos diferentes cenários de desenvolvimento. As primeiras discussões para definir as funções da APS iniciaram-se na década de 1900 após a instituição do seguro nacional de saúde inglês, o qual propôs a reestruturação do modelo assistencial segundo níveis de densidade tecnológica e custos de tratamento, baseados nos atributos do primeiro contato, da integralidade, da coordenação da atenção e da longitudinalidade^(1)^.

No Brasil, a principal expressão da APS é a Saúde da Família (SF) que, em 2020, acompanhava 133.710.730 pessoas em todas as regiões, com 62,62% de cobertura populacional, contando com 43.286 equipes, além das unidades básicas de saúde que oferecem cuidados em saúde para o restante da população. A SF contribui para o acesso universal à saúde e busca implementar os atributos da APS por meio da atuação de uma equipe multiprofissional em ações de promoção, prevenção, reabilitação e vigilância em saúde nos territórios com populações adscritas^(2)^. A organização, a expansão e a consolidação da SF como principal representação de uma APS sólida pode ocorrer de distintas formas e magnitude nos municípios, dependendo do porte populacional, do índice de desenvolvimento humano^(3)^ e do interesse político. A APS integra um sistema de saúde constituído pelos níveis secundário e terciário com ações e serviços de média e alta densidade tecnológica. Nesse sistema, o encaminhamento para outros níveis de atenção tem relação direta com a continuidade do cuidado (COC)^(4)^.

A longitudinalidade deriva de longitudinal que significa lidar com o crescimento e as mudanças de indivíduos ou grupos no decorrer de um período de anos. Pressupõe uma relação pessoal de longa duração entre uma mesma equipe de saúde e usuários dos serviços que pode sofrer interrupções por qualquer motivo, sem descontinuar essa relação. Permite que a equipe desenvolva uma certa familiaridade com usuários e suas famílias a qual facilita: reconhecimento de problemas de diversas ordens, diagnóstico precoce de doenças, na atenção preventiva, assertividade no diagnóstico clínico, na prescrição medicamentosa e no acompanhamento de rotina. Concomitantemente, pode contribuir para: redução de internações hospitalares e custos globais de tratamentos; e aumento da satisfação e confiança dos usuários com a COC^(1)^.

Contemporaneamente no Brasil, os termos COC e vínculo longitudinal têm sido usados como sinônimos de longitudinalidade. Eles possuem sentidos semelhantes, embora constituam expressões diferentes^(5)^. No início dos anos 2000, foi proposta uma definição conceitual hierárquica da COC, considerando os registros dos usuários, os tipos e a localização dos estabelecimentos prestadores de ações e serviços, as relações interpessoais entre os profissionais e os usuários, os membros da familia e todos os envolvidos na produção do cuidado^(6)^. Dessa forma, estabeleceu-se que a COC admite a continuidade informacional (coleção organizada, sistêmica e disponível de informações médicas e sociais dos usuários), longitudinal/cronológica (local de referência onde tradicionalmente se produz e se recebe os cuidados) e interpessoal (relação de confiança e responsabilidade entre usuários e profissionais). Dito de outra forma, o mínimo de continuidade informacional é necessário para que a continuidade cronológica esteja presente mediante a continuidade interpessoal^(6)^. Nesse contexto, a expressão vínculo longitudinal é definida como uma relação terapêutica estabelecida entre usuários e profissionais de uma equipe em uma unidade de saúde, sendo esta uma fonte regular de oferta de cuidados ao longo do tempo^(7)^.

Há três dimensões para o atributo da longitudinalidade^(7)^: a existência e o reconhecimento de uma fonte regular de cuidados primários, o estabelecimento de vínculo terapêutico duradouro entre os usuários e os profissionais de uma equipe e a continuidade do registro de informações sobre o problema de saúde do usuário do serviço. Observa-se que essas dimensões estão alinhadas aos elementos constitutivos da COC de estudo dos Estados Unidos^(6)^ o qual propôs: continuidade longitudinal/cronológica, interpessoal e informacional, respectivamente.

Internacionalmente, diversos instrumentos são utilizados para avaliar a COC: índice de continuidade dos cuidados, número de prestadores utilizados, índice de continuidade sequencial, índice de probabilidade de continuidade, índice de probabilidade de continuidade sequencial, dentre outros^(6)^. No Brasil, a longitudinalidade e os demais atributos da APS têm sido avaliados por instrumentos específicos, a exemplo do *Primary Care Assessment Tool* para pacientes crianças e adultos, com o objetivo de avaliar diversos aspectos sob a ótica de quem recebe o cuidado. Estudos transversais e populacionais já aplicaram essa ferramenta quantitativa, em diversas regiões, tanto em unidades de saúde da família (USF) quanto em unidades básicas de saúde^(3,8,9)^.

Em municípios do Rio Grande do Sul^(3)^ e Minas Gerais^(8)^, os usuários avaliaram a longitudinalidade como frágil sob o aspecto da continuidade interpessoal relacionada respectivamente: à falta de reconhecimento da integralidade do usuário que provocou uma produção limitada de cuidados e à falta de seguimento com os mesmos profissionais de uma equipe que interferiu no desenvolvimento da confiança entre eles. Em município do estado de Mato Grosso^(9)^, o atributo foi avaliado de maneira satisfatória, evidenciando a COC por meio da construção de vínculos terapêuticos duradouros entre usuários e profissionais.

Compreendendo que a longitudinalidade perpassa a COC, sendo um atributo essencial para o desenvolvimento e a consolidação de uma APS forte e estável, o presente estudo propõe-se a conhecer como os usuários apreendem a longitudinalidade e o que representa a COC na manutenção de sua saúde. Dessa forma, a dimensão qualitativa, explorando as vivências dos usuários do presente estudo, é que o diferencia daqueles que estudaram o tema por meio de instrumentos de avaliação. Assim, o objetivo do estudo é analisar a longitudinalidade na produção do cuidado na SF sob a ótica dos usuários.

## MÉTODO

### Tipo e Local de Estudo

Trata-se de estudo descritivo e analítico de abordagem qualitativa, inspirado nos critérios do instrumento denominado C*onsolidated Criteria for Reporting Qualitative Research*, realizado com usuários de duas equipes de SF (eSF) do município de Ribeirão Preto-SP, com 703.293 habitantes, dos quais apenas 165.600 ou 25% da população são acompanhadas por 51 eSF, distribuídas em 22 USF, sendo os demais acompanhados por equipes de Atenção Básica^(10)^.

### População e Critérios de Seleção

Inicialmente, contatou-se a coordenação municipal da APS, solicitando a indicação de duas eSF organizadas em sua configuração mínima com seus integrantes (médico, enfermeira, técnicos/auxiliares de enfermagem e agentes comunitários de saúde), atuando juntos há pelo menos dois anos, uma vez que esse é o tempo médio de permanência dos profissionais em uma mesma equipe^(11)^. Feita a indicação de três eSF, uma de essas equipes negou-se a participar no estudo, alegando reestruturação de seus integrantes e as outras duas aceitaram participar, sendo acolhidas como cenário da investigação. Essas eSF estavam alocadas em uma mesma USF e foram instruídas sobre o processo de desenvolvimento da pesquisa. No período de sua realização, cada eSF estava atuando com quatro agentes comunitários de saúde (ACS) e um ACS de cada eSF estava afastado de suas funções laborais com suas respectivas microáreas descobertas.

Para contemplar toda a área de abrangência da USF, foi solicitado a cada um dos oito ACS da USF a indicação de dois usuários de sua área e um de cada microárea descoberta, observando como critério de inclusão: adultos maiores de 18 anos em condição cognitiva de responder às perguntas, os quais fizessem seguimento com a equipe há pelo menos cinco anos^(1)^, tempo suficiente para os usuários estabelecerem vínculos longitudinais com a equipe na APS. Desse modo, a seleção dos participantes ocorreu por amostragem por conveniência, não havendo qualquer recusa na participação das entrevistas.

### Coleta de Dados

Foram realizadas duas abordagens com os usuários indicados pelos ACS: um contato telefônico para convite e agendamento da entrevista e o segundo para realização da entrevista em seus domicílios ou na USF, de acordo com sua preferência. As entrevistas semiestruturadas foram realizadas pela primeira autora durante o mês de maio de 2023, nos períodos matutino e vespertino, em dias úteis da semana, com duração máxima de 28 minutos. As entrevistas foram gravadas mediante autorização e norteadas por guia que investiga as dimensões da longitudinalidade no Brasil^(7)^, apresentado no Quadro 1. As perguntas do guia sofreram pequenas adaptações de linguagem para favorecer a compreensão dos usuários, por exemplo: “1.O senhor acha que a unidade tem uma população adscrita ou uma população para atendimento dentro de uma região?” O conteúdo das entrevistas foi suficiente para atender os objetivos do estudo, não sendo necessário incluir mais participantes.

**Quadro 1:** Questões para investigação das dimensões do atributo da longitudinalidade no Brasil^(7)^ – Rio de Janeiro, 2011.

Identificação ou reconhecimento da fonte regular de cuidados
1. A unidade possui adscrição de clientela? 2. A unidade é reconhecida pela população adscrita como local de atenção para antigos e novos problemas de saúde?
**Relação interpessoal**
3. O paciente é atendido regularmente pelo mesmo médico e/ou enfermeiro nas consultas de rotina? 4. Os profissionais que atendem o paciente conhecem o histórico familiar e social do paciente? 5. No caso de dúvida sobre tratamento, o paciente consegue falar com o profissional que o atende regularmente? 6. Durante o atendimento, há tempo suficiente para que os pacientes explicitem suas dúvidas, queixas e preocupações? 7. As dúvidas, preocupações e queixas do paciente são valorizadas? São registradas no prontuário? 8. O profissional se expressa com clareza, de forma que o paciente entenda? 9. Há espaço para que o paciente discuta seu tratamento e tome decisões junto com o profissional?
**Continuidade informacional**
10. Os profissionais de saúde utilizam o prontuário nos atendimentos? 11. Os profissionais de saúde estão informados sobre todos os medicamentos utilizados pelo paciente? 12. Os profissionais de saúde estão informados sobre os exames realizados pelo paciente? 13. Em caso de encaminhamento para especialista, este profissional recebe informações registradas da unidade que atende o paciente regularmente? 14. No caso de referência para consulta com especialistas ou exames externos, há retorno dos resultados para o clínico que atende o paciente regularmente? 15. Os profissionais de saúde são informados quando um paciente não consegue obter o medicamento prescrito?

**Fonte:** Estudo nacional^(7)^.

### Análise e Tratamento dos Dados

As entrevistas foram transcritas por empresa especializada e seu conteúdo, após conferido, foi analisado na seara de relações entre as partes, interpretando os sentidos das falas para compreender a visão dos usuários sobre o tema da pesquisa, ancorada pelo referencial metodológico da interpretação dos sentidos. Este referencial insere-se dentre as correntes compreensivistas das ciências sociais – que se dedicam a análise de palavras, grupos, instituições, contextos e suas relações – e avança na interpretação para além dos conteúdos, considerando o contexto e a lógica produzidos em uma determinada cultura e sociedade^(12)^. Foi desenvolvido em três etapas: exploração do material com a impregnação dos depoimentos; definição de ideias buscando revelar o implícito além do explícito, interpretando-as e problematizando-as^(12)^ e busca por sentidos mais amplos que articularam o objetivo do estudo, o referencial teórico-conceitual e os dados empíricos. Para a apresentação das ideias extraídas da análise do material empírico foi construída uma matriz interpretativa retomando os elementos constituintes da COC e as dimensões da longitudinalidade na APS a fim de ressaltar aproximações e afastamentos, sobretudo no sentido que englobou uma quantidade maior e mais diversificada de ideias, conforme compartilhado no Quadro 2 na seção dos resultados.

**Quadro 2:** Matriz interpretativa da pesquisa de acordo com referencial metodológico^(12)^, contendo os três sentidos mais amplos e suas respectivas ideias.

Organização e operacionalização do trabalho na USF
1. Área de abrangência da USF
2. Limitação da capacidade de atendimento da USF
3. Processo de trabalho na USF
4. Função do ACS
5. Atendimento longitudinal da equipe
6. Instrumentos de trabalho (prontuário)
7. Rotatividade profissional na equipe
8. Prioridade no atendimento na USF
9. Olhar centrado na doença para produção do cuidado
10. Olhar ampliado para produção do cuidado
11. Falta de intimidade dos profissionais com o usuário
12. Confiança e vínculo
13. Empatia e diálogo
14. Comunicação assertiva
15. Qualidade de atendimento na USF
**Organização e operacionalização do cuidado de si**
16. Corresponsabilidade do usuário pela sua saúde
17. Conhecimento do usuário sobre sua saúde
**Organização e operacionalização do sistema de saúde**
18. Referência e contrarreferência
19. Limitação da capacidade de atendimento do nível secundário
20. Organização do sistema de saúde
21. Responsabilidade estadual relacionada a medicamentos de alto custo
22. Limitações da oferta de medicamentos essenciais

**Fonte:** Elaborado pelas autoras.

### Aspectos Éticos

O estudo respeitou as normativas éticas das pesquisas com seres humanos seguindo a Resolução nº 466/2012, sendo aprovado por Comitê de Ética em Pesquisa, sob parecer número 5.933.046/2023. Todos os participantes assinaram previamente o Termo de Consentimento Livre e Esclarecido. Visando a preservação de anonimato, os participantes foram identificados a partir de um código alfanumérico (letra E seguida por número ordinal).

### Disponibilidade de Dados

Todo o conjunto de dados anonimizados que dá suporte aos resultados deste estudo foi disponibilizado, em fevereiro de 2024, no repositório de dados de pesquisa *ScIELO Data*, cuja versão preliminar está disponível em: https://data.scielo.org/dataset.xhtml?persistentId=doi:10.48331/scielodata.6JB0YH.

## RESULTADOS

Dos 18 participantes, 14 foram mulheres e 04 homens, com idade entre 30 e 87 anos. Quanto ao grau de instrução, oito possuíam primeiro grau incompleto, cinco possuíam primeiro grau completo, quatro possuíam segundo grau completo e uma possuía terceiro grau completo. Metade dos participantes referiu ser aposentado ou pensionista, sete são trabalhadoras do lar, um referiu ser porteiro e uma era assistente de recursos humanos. A maioria reside na área de abrangência da USF há mais de dez anos, uma há menos de dez anos e a outra não soube dizer.

Após a análise do material empírico, 22 ideias sobre a longitudinalidade na produção do cuidado na SF foram identificadas e agrupadas em três sentidos mais amplos, ordenados segundo uma lógica de entendimento e conformação das condições essenciais para fortalecer e garantir a COC e a longitudinalidade na APS relacionados respectivamente à: organização e operacionalização do trabalho na USF, do cuidado de si e do sistema de saúde, conforme o Quadro 2.

### Organização e Operacionalização do Trabalho na USF

No primeiro sentido, os entrevistados compartilharam elementos a respeito das restrições organizacionais (área de abrangência e limitação da capacidade de atendimento médico), da força de trabalho (composição e atribuição dos membros das eSF, suas competências e habilidades para produzir o cuidado), das tecnologias duras (instrumentos para a produção do cuidado) e das tecnologias leves (confiança, vínculo, empatia, diálogo, comunicação assertiva e falta de intimidade com o usuário):

*Eu acho que eles têm uma área de abrangência, porque eu já vi muita gente perguntar que queria ser atendido e falar que não podia, porque tem que estar localizado no bairro, então eles têm uma área que eles atendem (E16).*

*Essa questão de agenda aberta também é um pouco complicada para conseguir marcar uma consulta (médica). Porque, na maioria das vezes, eles falam que não têm agenda aberta, então você tem que ficar indo todo dia para ver se tem uma agenda aberta (...). Eu fui marcar com a (nome da enfermeira), foi muito rápido. Para fazer o Papanicolau foi muito rápido. Mas com ela (nome da médica), é um pouquinho mais difícil. Porque ela atende muita gente. É muito paciente. É assim, você chega lá, está sempre cheio, lotado (E10).*

*A gente tem que passar para pesar, medir, ver a pressão (E5).*

*As meninas do atendimento, da pré-consulta, você conversa com elas. Se eu precisar falar com ele (o médico), eu passo por elas. Talvez elas consigam falar com ele, me dão um retorno, entendeu? (E15).*

*A enfermeira, você chega lá, ela além de te pôr muito à vontade para você falar tudo o que você tem direito de falar... Quer dizer, você tem um jeito de falar tudo o que você quer para ela e contar tudo, até particulares. E ela te recebe muito bem e não fala: Vamos, que tem outra paciente esperando e tal.... Então ela te dá todo o amparo no tempo que você precisa. Por isso que ela marca um tempo já bem diferenciado para cada um (E8).*

*Todas as vezes que a gente vai fazer uma consulta, ele (o médico) pega o prontuário todo e lê para a gente ali (E9).*

*A gente costuma conversar não só os problemas de doença, você tem um filho que está doente ou algum desempregado, eu tenho um filho que faz uns três anos que descobriu que estava com câncer, então se você perguntar para a doutora, ela sabe contar a história nossa... você começa a ter um atendimento familiar mesmo, ela sabe das coisas. Se ela vem aqui em casa, ela já pergunta de todo mundo, ela sabe o nome dos meus filhos, então eu acho que não é só o físico, ela tem uma convivência familiar (E16).*

*Ah, eu discuto, eu pergunto. Que nem: a psicoterapia. Eu falo: Por que que você acha que eu preciso fazer? É porque você está com aquele probleminha de ninho, como que é? Abandono do ninho... Síndrome do ninho vazio. Eu estou com isso, porque os filhos foram embora, não tem ninguém aqui mais, só eu e essa irmã. Ela (a médica) sentiu a hora que eu fui falar sobre os filhos, ela sente que eu fico meio magoada, porque eu estou aqui sozinha, mas não é culpa deles... Aí a doutora sabe tudo e ela falou: Eu vou te mandar para lá, você aceita? Ela não me força a nada. Ela me deu um papel, eu levei lá no balcão e já comecei. Quer dizer. Ela me entende (E2).*

*Elas já aprenderam a nossa língua do bairro aqui, elas falam nossa língua, então dá para entender, eu a elas; elas, a mim (E2).*

*O doutor Z era um médico maravilhoso, é um tipo (nome da enfermeira). Que absorvia os seus problemas até particulares e contava os dele também, que é a coisa mais difícil do mundo. O doutor Y, se não olhar aqui na recepção, você não sabe nem o nome dele direito; e aquele não (...). Ele conhecia cada paciente pelo nome. Já o que não acontece atualmente, você entendeu? (E8).*

Neste sentido houve uma aproximação maior com a continuidade interpessoal ou estabelecimento de vínculo terapêutico duradouro entre profissionais das eSF e usuários e com a continuidade longitudinal/cronológica ou existência e reconhecimento de uma fonte regular de cuidados primários. Esses elementos e dimensões foram vivenciados respectivamente pelos usuários na medida em que identificaram integrantes das eSF como sujeitos referência para a implementação de práticas de saúde necessárias e oportunas, sendo a USF o local determinado para esse fim.

### Organização e Operacionalização do Cuidado de si

No segundo sentido, os entrevistados destacaram sua corresponsabilidade pela sua condição de saúde e estilo de vida, enfatizando o autocuidado (prática e manutenção de hábitos saudáveis), o autoconhecimento (sobre seus agravos e possíveis complicações) e a socialização, tal como indicam os fragmentos:

*Eu pertenço a um grupo de pessoas, de até 97 anos que pratica esporte. É corrida e lançamento de peso que eu faço. Eu treino duas a três vezes por semana. Talvez dia 20 nós vamos lá para (nome da cidade) disputar. Tem várias coisas, mas no meu caso é correr e arremesso. Então a gente faz isso aí: treina bastante lá. Eu encosto quase a palma da mão no chão, com as pernas esticadas. Antes de fazer qualquer coisa, a gente faz um bom tempo de ginástica, para depois ir treinar, correr, uma série de coisas (E4).*

*Eu tomo remédio só para tireoide, não tenho problema de pressão; a pressão é 11 por 8, 12 por 8. Eu não deixo subir. Papanicolau, mamografia, eu já corro atrás, ele (o médico Z) manda eu fazer, eu já vou fazer (E9).*

*Antes da pandemia, a gente fazia festa, era Dia das Mães, Dia dos Pais, Festa Junina (...). É festa de fim de ano, confraternização, aí arruma um clube, a gente passa o dia no clube, faz amigo secreto... Quem vai, leva um presente. Não é coisa cara, é uma lembrancinha. Aí já faz na hora os papeizinhos, sorteia e a gente faz, passa o dia. Cada um leva um prato, torta, bolo, o que quiser. Com o pessoal do posto, a gente tem isso tudo (...). A gente faz churrasco, cada um leva uma coisa e passa o dia no clube (E5).*

Neste sentido, salientou-se a postura ativa dos usuários no gerenciamento da própria vida em busca do bem-estar e da qualidade de vida, assumindo a responsabilidade pelo autocuidado associada à relevância do convívio social facilitado pelas ações extramuros organizadas pelas eSF, ideia que converge à continuidade interpessoal ou estabelecimento de vínculo terapêutico duradouro entre profissionais e usuários da USF. Ao mesmo tempo, infere-se que nesse sentido, o elemento e a dimensão acima trazem implicitamente uma relação próxima à existência e reconhecimento de uma fonte regular de cuidados primários visto que diversos encontros grupais são organizados para compartilhar datas festivas durante um ano para auxiliar na promoção de saúde.

### Organização e Operacionalização do Sistema de Saúde

No terceiro sentido, os entrevistados compartilharam seu entendimento sobre o funcionamento dos três níveis de atenção, reconhecendo suas demarcações estruturais e tecnológicas (referência e contrarreferência, limitações da capacidade de atendimento secundário associadas a restrições do sistema informatizado e da oferta de medicamentos básicos e especializados) respectivamente evidenciado nos fragmentos:

*Você pode ser tratado (na USF) porém, se for alguma emergência, a gente sempre tem a orientação de procurar a UPA (Unidade de Pronto Atendimento), mas aqui a gente tem consultas (...) um diabetes que vai tratar, a médica da equipe vai dar medicamento, um tratamento futuro (...) (E11).*

*Se aparecer um caso novo (eles tratam). Aí se eles não puderem tratar é encaminhado para o médico específico, por exemplo, se precisar ir para ortopedia, neuro, geriatra. Então, os casos novos que aparecem eles tentam dar solução, se eles não podem tratar, eles encaminham para o lugar que... para área específica (E16).*

*Se eles (os médicos que atendem no nível secundário) mandam retorno para doutora (a médica da eSF) eu nunca ouvi falar nada, não. Eu acho que não. Se eu trato lá, depois continua lá, não sei se depois vai voltar para cá, dá um papel só, sei lá (E6).*

*Eu estou precisando de oftalmo e não é de hoje que eu estou querendo marcar. Não consigo marcar. Me deram o telefone (...). Uma hora está desligado, uma hora está ocupado, outra hora chama e não atende (...). Eu não vou no oftalmo faz uns 2 anos e meio. Eu já estou sentindo, porque eu dirijo e à noite eu não estou conseguindo dirigir mais, porque os óculos já não estão prestando muito (E2).*

*Os profissionais aqui trabalham no topo já, porque às vezes não há condições mesmo de... eles recebem para passar para nós. Tudo o que pode ser feito por eles é feito (...). Às vezes, por exemplo, não tem um remédio no posto por quê? Porque a Secretaria da Saúde não mandou esse remédio, entendeu? (...) Esse posto aqui faz tudo o que pode para mim. Se não tem um remédio aqui, eles ligam para o outro posto para eu ir buscar (E12).*

*Acontece de não encontrar o remédio, que tem uns tipos de remédio que eles fazem um documento para buscar no Hospital das Clínicas. É um... não sei o que lá de alto preço (...). Eu fui lá e não tinha o remédio que estava no documento. Quase 20 dias depois, já tinha o remédio que a gente liga para saber se tem (E4).*

Neste sentido, os dados empíricos indicam que os usuários da USF têm consciência sobre a organização do sistema de saúde em níveis de atenção, associando essa vivência à continuidade longitudinal/cronológica ou à existência e reconhecimento da USF como fonte regular de cuidados primários, descrevendo dificuldades para acessar outros serviços para os quais são encaminhados oportunamente. Ademais, atestam a responsabilidade da esfera municipal na oferta e disponibilidade de determinados produtos essenciais à saúde. A continuidade informacional ou a continuidade do registro de informações sobre o problema de saúde do usuário parece se limitar a cada um dos serviços utilizados, não havendo articulação dos dados clínicos entre eles.

## DISCUSSÃO

A caracterização sociodemográfica dos entrevistados, com uma maioria constituída por mulheres e idosos com baixo nível instrucional em ocupação informal, reflete o índice de transição demográfica para cada segmento social desde os anos 1980 até 2010, determinado por um diferencial no gênero da esperança de vida ao nascer atribuído ao aumento de óbitos por causas violentas que acometeram majoritariamente os homens. Além disso, um perfil etário mais envelhecido com redução dos níveis de fecundidade, devido às transformações socioeconômicas brasileiras, influenciou diretamente o comportamento reprodutivo feminino. E ainda, um novo perfil de pessoas foi incorporado ao atual mercado consumidor: a classe média baixa com renda de até dois salários-mínimos – consumidora massiva de ações, bens e serviços – graças a políticas públicas sociais inclusivas voltadas para a transferência de renda^(13)^.

A esse cenário da transição demográfica nas últimas três décadas, soma-se a trajetória do envelhecimento populacional que até o ano de 2030 atingirá a razão de 76 idosos para cada 100 jovens. Essa projeção desperta a discussão sobre a atenção à saúde que implicará no redimensionamento da oferta de ações e serviços desse setor – em sua maioria ainda voltadas para o atendimento de condições agudas e agudizações das condições crônicas – considerando os agravos crônicos não transmissíveis da população idosa, bem como os custos associados a seus respectivos tratamentos^(13)^. Nessa lógica de produção de cuidados que polariza os eventos de saúde, destaca-se a potência da longitudinalidade, desenvolvida pelas eSF, que pode sustentar um acompanhamento mais eficaz, efetivo e eficiente do conjunto de morbidades inerentes a esse segmento populacional que a cada ano avança em longevidade.

Quanto à organização e operacionalização do trabalho na USF, as restrições da oferta de atendimento médico apontadas pelos usuários também foram evidenciadas em outros estudos^(8,14-16)^ que destacaram a demora entre o agendamento da consulta médica e o atendimento em si. Esse fato pode implicar em complicações das condições crônicas de saúde dos usuários, uma vez que consultas regulares e frequentes na APS representam o acesso e a continuidade da produção dos cuidados pela equipe, tendo relação direta com: satisfação dos usuários com o serviço, sua qualidade de vida, adesão aos tratamentos propostos, indicadores de saúde, redução de custos de hospitalização^(17)^ e menos encaminhamentos para outros níveis de atenção^(4)^.

O usuário reconhece o protagonismo do trabalho da enfermeira enquanto organizadora da produção do cuidado na USF, que envolve o planejamento e a definição do acesso dos usuários às ações e serviços necessários para cada caso promovendo a COC. Essa postura ativa fortalece o elemento da continuidade interpessoal^(6)^ ou o estabelecimento de vínculo terapêutico duradouro entre os profissionais e usuários^(7)^, resolve conflitos e conduz situações no cotidiano assistencial, influenciando diretamente o processo de trabalho da equipe^(14,18,19,)^.

O grau de afiliação dos usuários também está centrado no médico, que é reconhecido como seu profissional de referência^(9,19)^. Todavia, os usuários que não identificam o nome do médico que os acompanham na APS podem desenvolver relações interpessoais frágeis com a equipe capazes de interferir no acesso e na continuidade da produção de cuidados^(20)^.

Apesar de alguns usuários referirem que a valorização dos aspectos biopsicossociais (capilarizados pelas tecnologias leves) contribuem para uma produção de cuidado mais ampliada, a tríade queixa-diagnóstico-conduta também é criticada e retratada por meio da falta de diálogo e engessamento de ações curativistas^(16,19,21)^. No universo das tecnologias leves, a clareza das orientações técnicas transmitidas pelos profissionais de saúde também configura uma comunicação assertiva, um dos elementos constituintes de uma longitudinalidade efetiva^(16)^.

Os usuários parecem saber que existe uma tecnologia dura que organiza e sistematiza seus dados clínicos para a eSF e demais profissionais de saúde de outros níveis de atenção prescreverem o cuidado, entretanto, não demostraram consciência da relevância do fluxo dessas informações entre os serviços. O uso de prontuário físico vem sendo substituído por sistemas informatizados que permitem acesso a informações administrativas, sociais e clínicas dos usuários, mesmo que estejam fragmentadas ou que tenham sido produzidas por distintos profissionais e prestadores de serviço. Para que essa tecnologia dura expresse o elemento da continuidade informacional^(6)^ ou a dimensão da continuidade do registro de informações sobre o problema do usuário^(7)^ é necessário organizar os dados de forma que estejam disponíveis oportunamente para as equipes dos diferentes níveis de atenção produzirem cuidados mais ampliados, articulados e integrais. Em contrapartida, diferentes sistemas operacionais utilizados por distintos serviços de saúde que não se conectam dificultam o desenvolvimento da longitudinalidade da atenção^(17,18,22,23)^, sobretudo para portadores de condições crônicas de saúde que demandam maior regularidade assistencial^(24,25)^.

Segundo os usuários, a organização do fluxo assistencial no interior do serviço ocorre de forma limitada, levando à segmentação do atendimento^(16)^, oportunidade em que cada profissional utiliza sua expertise técnica e científica, registra as informações no prontuário e encaminha o usuário para o próximo profissional que agregará seus conhecimentos de maneira semelhante, até a chegada do médico – prática comum nas USF do município cenário da investigação, fruto da divisão técnica e social do trabalho em saúde.

Sobre o elemento da continuidade interpessoal ou a dimensão do estabelecimento de vínculo terapêutico duradouro entre profissionais e usuários reproduzidos pelas tecnologias leves, os usuários evidenciaram que a atitude acolhedora da equipe expressa por cordialidade, escuta ativa, diálogo, atenção, empatia, afeto e responsabilização contribuem para a construção de laços entre usuários e trabalhadores, promovendo a satisfação dos usuários com o trabalho oferecido na USF e a adesão aos planos terapêuticos para controle das condições crônicas de saúde^(15,26)^. A confiança dos usuários na equipe é um conceito essencial que predispõe a um maior envolvimento e comprometimento pessoal com sua própria saúde^(27)^. Essas habilidades podem favorecer a familiaridade dos integrantes da equipe com os usuários e suas famílias, na medida em que se mostram disponíveis em conhecer seus problemas e angústias, responder dúvidas e necessidades^(27)^. Portanto, partindo da premissa da criação de um vínculo por meio de fortes laços interpessoais e cooperação, a concretização de um cuidado longitudinal, nas diferentes fases da vida, permite valorizar a concepção ampliada do processo saúde-doença e gerar satisfação com o atendimento recebido na APS^(21)^.

A respeito da organização e operacionalização do cuidado de si, a corresponsabilização do usuário pela sua condição de saúde e estilo de vida pode ser associada à produção de uma longitudinalidade qualificada de duas formas: a primeira caracteriza-se por uma resposta a uma demanda pontual ou específica traduzida por uma longitudinalidade descontínua ou focalizada que se constitui no elemento da continuidade longitudinal/cronológica^(6)^ ou na dimensão da existência e reconhecimento da USF como uma fonte regular de cuidados primários^(7)^ buscada a cada novo evento ou problema de saúde. A segunda caracteriza-se por uma longitudinalidade contínua ou ampliada expressa por uma resposta frequente, duradoura e segura para o controle, por exemplo, de uma condição crônica de saúde que depende da construção do elemento da continuidade interpessoal^(6)^ ou da dimensão do estabelecimento de vínculo terapêutico duradouro com a eSF^(7)^.

A concepção da produção de cuidados ampliados e focalizados relacionados aos atributos da APS, foi inicialmente discutida^(28)^ na primeira década dos anos 2000, à época da implementação mais rápida e abrangente da SF pelo Brasil. Esse debate gerou uma classificação ao atributo da integralidade da atenção considerando seus diferentes sentidos^(28)^, os quais foram amplamente estudados na prática assistencial das eSF. Nesta segunda década de desenvolvimento moroso da SF, fruto de diversos movimentos de desmonte da APS e do Sistema Único de Saúde como um todo, apresentar uma qualificação para outro atributo, o da longitudinalidade, fundamentada em referencial teórico-conceitual internacional^(6)^ e nacional^(7)^, pode ser considerada um ato de resistência à produção de cuidados primários e à credibilidade do processo de trabalho das eSF.

Ainda no dispositivo do autoconhecimento e autocuidado, de um modo geral, as USF desenvolvem algum tipo de atividade coletiva associada à educação em saúde organizada pela equipe, tais como palestras, atividade física, dança, jogos, artesanato, coral ou outras que possibilitam o convívio social e a ocupação dos idosos^(15)^, uma vez que grande parte deles não possui rede de apoio ou família presente. No cenário internacional, iniciativas coletivas e individuais semelhantes que estimulam, conduzem e orientam singularmente a mudança de hábitos dos usuários da APS também são desenvolvidas por equipes multidisciplinares^(27)^. Esse movimento pode ser comparado ao trabalho dos Núcleos de Apoio à Saúde da Família que, integrados às eSF, focam em ações de promoção de saúde e prevenção de agravos – embora tenham perdido espaço com a reorganização do financiamento da APS^(29)^.

Ações de promoção de saúde com foco no envelhecimento ativo e saudável e organização de atividades coletivas extramuros, referidas pelos entrevistados, possibilitam a troca de experiências, atenuam o isolamento social e fortalecem laços interpessoais. Assim, coloca-se em prática um componente importante da APS: a produção do cuidado mediante um olhar ampliado do processo saúde-doença que ocupa espaço no modelo hegemônico centrado na doença^(25)^. Ademais, a valorização da autonomia e da participação ativa dos usuários na tomada de decisões relacionadas a sua saúde também contribuem para o fortalecimento do elemento da continuidade interpessoal ou da dimensão do estabelecimento de vínculo terapêutico duradouro entre usuários e profissionais contribuindo com a COC e longitudinalidade^(27)^.

Quanto ao terceiro sentido, da organização e operacionalização do sistema de saúde, as demarcações estruturais e tecnológicas reconhecidas pelos usuários podem ser comparadas a outras pesquisas na medida em que a contrarreferência também se mostrou frágil, não ocorrendo o retorno ao nível primário, do processo de atenção do usuário encaminhado^(14,18,21,22,30)^. Nessa realidade infere-se uma ruptura do elemento da continuidade informacional^(6)^ ou da dimensão da continuidade do registro de informações sobre o problema de saúde do usuários^(7)^.

Esse modo de funcionamento da referência e contrarreferência intensifica a fragmentação do cuidado e sugere uma baixa responsabilização dos profissionais dos diferentes níveis de atenção, comprometendo sua efetividade. Adicionalmente, a falta de vagas nos níveis secundário e terciário gera cuidados isolados e sem interação, mantendo o foco da atenção apenas na demanda que originou o encaminhamento^(8,14,18)^. E ainda, o encaminhamento para consultas especializadas, exames mais complexos e cirurgias é fruto da morosidade da rede de atenção à saúde^(8,15,22,30)^.

A falta de medicamentos foi apontada pelos usuários como um dos problemas da organização e operacionalização do sistema de saúde. Dessa forma, a falta de medicamentos essenciais é um aspecto que pode fragilizar a produção do cuidado e resultar em descrédito e insatisfação dos usuários com a USF e com o funcionamento da rede de saúde, fazendo-os buscar outros estabelecimentos de saúde para ter sua demanda atendida ou aguardar o restabelecimento local^(14-16)^. Essa situação pode contribuir com a falta de adesão ao tratamento proposto pela equipe, pois nem sempre os usuários têm condição financeira para comprar o medicamento prescrito, ainda que básico e estratégico^(31)^. Da mesma forma, o acesso do usuário ao Componente Especializado da Assistência Farmacêutica nos municípios, de responsabilidade da esfera estadual, é dificultado pela falta de medicamentos e de informação dos usuários, pela burocracia na dispensação e demora na aquisição de determinados fármacos^(32)^.

As limitações do estudo estão no fato de que os usuários entrevistados constituem os que mais frequentam a USF e mais cuidam de sua saúde, haja vista a população composta majoritariamente por mulheres, o que pode ter influenciado parte dos resultados. Ademais, os dados qualitativos retratam a realidade de duas eSF em um município com baixa cobertura da SF e alta rotatividade profissional, o que dificulta a generalização dos achados. Portanto, indica-se a realização de outros estudos que se proponham a analisar, qualitativamente, a COC e a longitudinalidade em outros cenários e contextos da APS.

Entre os benefícios desta investigação, incluem-se a identificação e a discussão sobre os elementos constituintes da COC e as dimensões da longitudinalidade sob a ótica dos usuários, de sua relevância e de sua operacionalização.

## CONCLUSÃO

Os usuários da SF reconheceram a longitudinalidade por meio de uma demarcação estrutural, tecnológica e organizacional, identificando a força de trabalho na produção de cuidados implementados por tecnologias leves e duras, compartilhando fragilidades e potencialidades. Admitem o que precisam, querem ou entendem como necessário para manter e tratar sua saúde, tanto com relação à organização do sistema como um todo, como em relação ao que se passa na USF e, mais particularmente, nas relações com a eSF ou algum trabalhador em específico e sua responsabilidade nesse processo.

A longitudinalidade é um atributo da APS em construção permanente para garantir a COC. Ela depende da organização macropolítica de uma rede de saúde com ações e serviços oportunos. Ao mesmo tempo, depende de encontros no trabalho vivo em ato, ou seja, na micropolítica de sua produção, pois quem operacionaliza a máquina denominada sistema de saúde são pessoas – trabalhadores e usuários.

A SF pode oferecer uma longitudinalidade contínua ou ampliada. Entretanto, uma baixa cobertura da estratégia pode comprometer o desenvolvimento desse atributo sob essa perspectiva na medida em que limita o acesso às ações, serviços e profissionais de saúde nos demais níveis de atenção e consequentemente, à COC. Como a COC e a longitudinalidade envolvem as continuidades informacional (ou do registro de informações sobre o problema do usuário), longitudinal/cronológica (ou existência e reconhecimento de uma fonte regular de cuidados primários) e interpessoal (ou estabelecimento de vínculo terapêutico duradouro entre profissionais e usuários), se torna imperioso desenvolver a continuidade de gestão desses processos que se entrelaçam na coordenação da rede de atenção à saúde, considerando as nuances do envelhecimento populacional.
